# Chitosan-Based Bioactive Hemostatic Agents with Antibacterial Properties—Synthesis and Characterization

**DOI:** 10.3390/molecules24142629

**Published:** 2019-07-19

**Authors:** Julia Radwan-Pragłowska, Marek Piątkowski, Volodymyr Deineka, Łukasz Janus, Viktoriia Korniienko, Evgenia Husak, Viktoria Holubnycha, Iryna Liubchak, Vyacheslav Zhurba, Aleksandra Sierakowska, Maksym Pogorielov, Dariusz Bogdał

**Affiliations:** 1 Faculty of Chemical Engineering and Technology, Cracow University of Technology, 31-155 Kraków, Poland; 2 Medical Institute, Sumy State University, Sumy 40007, Ukraine; 3 Osteoplant Research and Development, 39-200 Dębica, Poland

**Keywords:** hemostatic agents, antibacterial biomaterials, chitosan, polymeric materials, green chemistry

## Abstract

Massive blood loss is responsible for numerous causes of death. Hemorrhage may occur on the battlefield, at home or during surgery. Commercially available biomaterials may be insufficient to deal with excessive bleeding. Therefore novel, highly efficient hemostatic agents must be developed. The aim of the following research was to obtain a new type of biocompatible chitosan-based hemostatic agents with increased hemostatic properties. The biomaterials were obtained in a quick and efficient manner under microwave radiation using l-aspartic and l-glutamic acid as crosslinking agents with no use of acetic acid. Ready products were investigated over their chemical structure by FT-IR method which confirmed a crosslinking process through the formation of amide bonds. Their high porosity above 90% and low density (below 0.08 g/cm^3^) were confirmed. The aerogels were also studied over their water vapor permeability and antioxidant activity. Prepared biomaterials were biodegradable in the presence of human lysozyme. All of the samples had excellent hemostatic properties in contact with human blood due to the platelet activation confirmed by blood clotting tests. The SEM microphotographs showed the adherence of blood cells to the biomaterials’ surface. Moreover, they were biocompatible with human dermal fibroblasts (HDFs). The biomaterials also had superior antibacterial properties against both *Staphylococcus aureus* and *Escherichia coli*. The obtained results showed that proposed chitosan-based hemostatic agents have great potential as a hemostatic product and may be applied under sterile, as well as contaminated conditions, by both medicals and individuals.

## 1. Introduction

Restoration of hemostasis after injury is still an ongoing challenge despite the numerous researches in this area. Massive blood loss can be a consequence of a transport accident or may occur on the battlefield [1,2,3]. Hemorrhage appears also during surgeries and as a result of certain diseases [4,5,6,7]. Despite modern surgical techniques and new hemostatic materials, bleeding is one of the most common causes of death in surgery [2]. Application of traditional dressings with the purpose to stop a hemorrhage is effective only in the case of superficial wounds. Any skin tissue damage associated with excessive bleeding requires use of a hemostatic agent [7]. In ancient times to prevent massive blood loss various natural components were applied, such as herbs, waxes, grease and others. Currently there are a few types of hemostatic agents, such as physical agents, resorbable agents, biological agents, synthetic agents and dressings [1,2]. The most popular ones are biodegradable gelatin and collagen foams, fibrin sealants as well as chitosan granules, sponges and dressings [1,2,3,4,5,6,7]. Hemostatic agents may be used in hospitals and by individuals. Thus, the environmental conditions of their application can be very diverse. Some injuries may occur under non-sterile conditions [7,8,9]. Traumatic wounds which are exposed to dirt and pathogens are susceptible to infections. Therefore, an ideal hemostatic agent should have antimicrobial properties. This can be achieved by applying a dressing containing antibiotics or metallic nanoparticles that are effective against most bacteria species [8,9]. Nevertheless, in the case of some resistant populations commonly used biomaterials are insufficient. On other hand, chemical agents or nanoparticles can cause complications, including allergy or tissue damage [8,9].

A most promising class of biomaterials dedicated to hemostasis maintenance are materials which after contact with an aquatic medium become hydrogels. Their advantages include excellent water sorption properties, flexibility, durability and possibility incorporation of different bioactive substances. The most common are those based on biopolymers such as collagen, gelatin, hyaluronic acid, cellulose, heparin as well as synthetic ones such as poly(ethylene glycol). A good hemostatic agent should be characterized by a lack of cytotoxicity, biodegradability and a high rate of blood sorption. There are two main types of hydrogels depending on their preparation method. They may be obtained as a result of physical or chemical crosslinking. Physically cross-linked hydrogels are less durable and have a reversible nature which means that after pH or temperature change they may lose their integrity. Chemically cross-linked hydrogels are much more resistant to environmental conditions and preserve their mechanical properties for a longer period of time. On the other hand, chemical hydrogels can be toxic due to the application of certain crosslinking agents such as glutaraldehyde or photoinitiators [10].

Hydrogels can be modified with antibiotics, iodine ions or metallic nanoparticles to enhance their antimicrobial activity [8,9]. However, such additives may have a negative impact on their biocompatibility. Therefore, preparation of the efficient hemostatic agent with antibacterial properties is still an ongoing challenge. Chitosan is a natural polymer obtained from chitin during deacetylation process. It consists of two types of mers–*N*-acetylaminoglucose and aminoglucose. It is widely applied in medicine due to its superior biological properties such as cytocompatibility and anti-pyrogenic activity [11,12,13,14,15,16,17,18]. Moreover, due to the presence of free amino groups, chitosan has antibacterial properties against various bacteria strains [19,20]. Chitosan is also susceptible to biological degradation [21,22]. Nevertheless, pure chitosan has quite low durability and poor stability in aquatic media [19]. A unique feature of the biopolymer is its hemostatic [23,24,25] and antioxidant activity [26,27,28].

Chitosan due to its poly(cationic) nature has mucoadhesive properties and may interact with sialic acid—glycoprotein present in the mucus [29]. Presence of positively charged free amino groups coming from aminoglucose mers is also responsible for analgesic effects which occur due to ions being released in the inflammatory area. The biopolymer may lead to the reorganization followed by opening of the tight junction proteins which results in the membrane permeation increase [30,31]. Finally, chitosan can stop a hemorrhage due to the interactions with negatively charged thrombocytes and erythrocytes resulting in blood clot formation [32,33]. Thus, it constitutes a great candidate for the most efficient hemostatic agent with various bioactive properties.

The aim of the following research was to obtain novel chitosan derivatives in a chemical crosslinking process which would be characterized with hemostatic properties and antimicrobial activity. To enhance biopolymer properties chemical modification using two amino acids as crosslinkers was performed in the field of microwave radiation. The proposed modification strategy enabled complete elimination of acetic acid application during the synthesis. The crosslinking process occurrence was confirmed by infrared spectroscopy which showed new amide bonds formation. The samples had very high porosity and low density which positively affect chitosan aerogels sorption abilities. The obtained biomaterials exhibited excellent hemostatic properties and were non cytotoxic on human dermal fibroblasts. The blood clotting test showed that hemostatics were able to activate platelets. The microphotographs showed that all biomaterials were covered with blood cells after incubation. Moreover, chitosan derivatives had antibacterial activity against both gram negative and gram positive strains. Also, they had very good water vapor permeability which is crucial during wound recovery in the case of superficial bleeding. The biomaterials also exhibited the ability to remove free radicals. Finally, the hemostatic agents were biodegradable under human-like conditions, thus they may be applied during surgery or for internal bleeding without necessity of their removal after clot formation.

Overall, performed studies showed that proposed biomaterials have great potential in the field of advanced hemostatic agents since they can prevent massive blood loss and accelerate skin regeneration by promotion of fibroblast proliferation and new tissue formation.

## 2. Results

### 2.1. Fourier Transform Infrared Spectroscopy (FT-IR) Analysis

**Figure 1:**
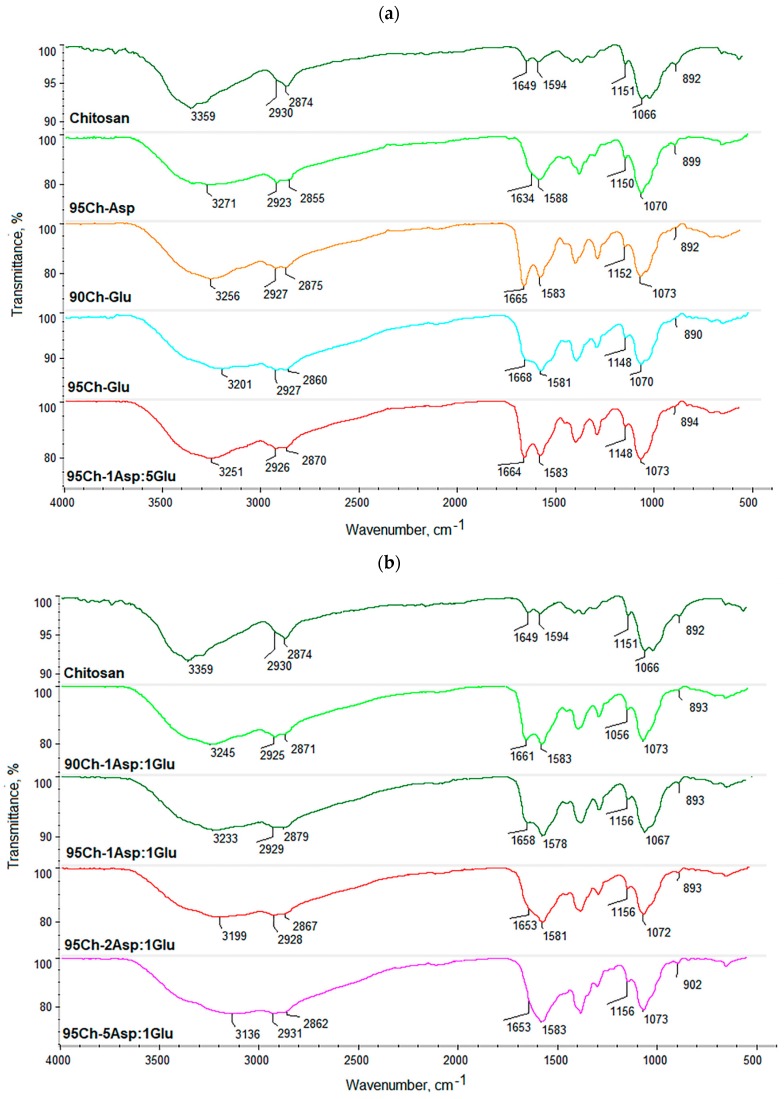
(**a**,**b**) FT-IR spectra of the pure chitosan and the aerogels prepared via chitosan crosslinking using glutamic and aspartic acid.

Figure 1 presents FT-IR spectra of the raw polymer and obtained biomaterials. Pure chitosan spectrum exhibits some typical bands coming from free hydroxyl at 3359 cm^−1^ as well as amino groups at 1594 cm^−1^ and 1151 cm^−1^ (deacetylated unit). Bands with the maximum at 2930 cm^−1^ and 2874 cm^−1^ correspond to aliphatic groups. Bands with the maximum at 1649 cm^−1^ are characteristic for amide bonds present in acylated units. A band at 1066 cm^−1^ comes from glycosidic bonds between chitosan mers, whereas a band at 893 cm^−1^ is typical for pyranose rings. FT-IR spectra of all the samples show changes confirming crosslinking process such as increased intensity of the bands typical for amide bonds and free amino groups coming from amino acids. It can be observed that a new band coming from carboxyl groups (3270–3136 cm^−1^) can be observed which could be formed as a result of slight surface degradation. Acidic groups may also come from grafted amino acids. Another significant change is an increased intensity of bands typical for amide bonds which proves the reaction between carboxylic groups coming from amino acids and free amino groups coming from chitosan (1668–1634 cm^−1^). At the same time, bands corresponding to free amino groups are still present (1588–1578 cm^−1^; 1156–1148 cm^−1^). Thus, it can be concluded that the proposed modification strategy resulted in the crosslinking of chitosan with simultaneous preservation of functional groups responsible for chitosan’s unique properties [34,35]. It can also be observed, that microwave radiation did not cause any significant polymer degradation since there are no changes in the bands coming from glycosidic bonds (1073–1067 cm^−1^) as well as pyranose rings (902–890 cm^−1^). The bands characteristic for aliphatic groups are also still visible (2931–2923 cm^−1^; 2879–2855 cm^−1^). Overall, it can be assumed that obtained biomaterials will maintain favorable features of chitosan and may be characterized by extraordinary biological properties due to the increased amount of free functional groups [13,35]. The results also show that it is possible to obtain crosslinked material without applying acetic acid which may have a cytotoxic effect on cells.

**Figure 2:**
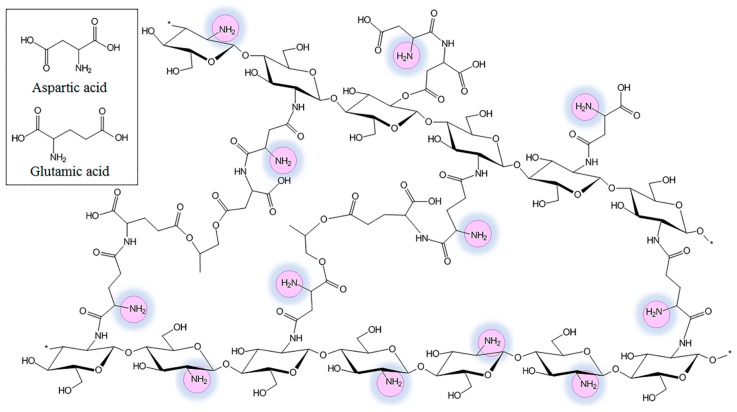
The proposed chemical structure of the crosslinked with glutamic and aspartic acid aerogels.

Basing on the data collected from the FT-IR spectra, biomaterials’ chemical structure was proposed as shown in the Figure 2.

### 2.2. Porosity and Density

**Figure 3:**
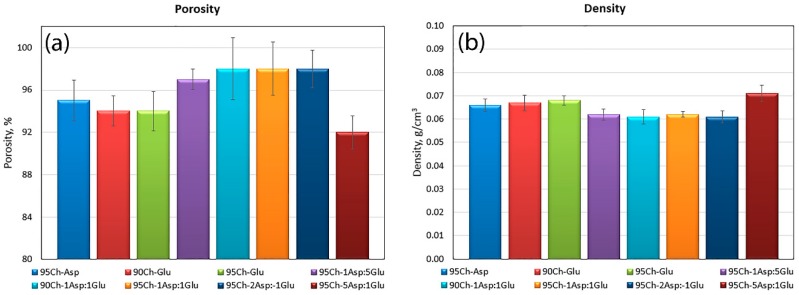
(**a**) Porosity and (**b**) density of the aerogels prepared via chitosan crosslinking using glutamic and aspartic acid.

**Figure 4:**
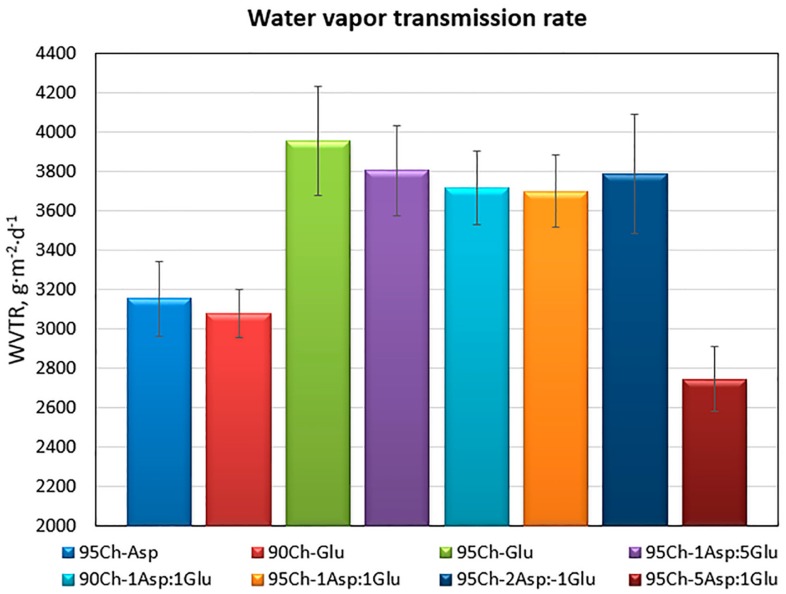
Water vapor transmission rate of the aerogels prepared via the crosslinking process using glutamic and aspartic acid.

It can be noticed that all of the samples have excellent porosity that is above 90% which suggests that the polymeric materials will be able to absorb a high amount of aquatic solutions and take part in blood clot formation (Figure 3) [35]. High porosity is an effect of the crosslinking process as well as lyophilization. The highest porosity occurred in samples containing both amino acids in their structure, however only until a certain point. The lowest porosity (95Ch-5Asp:1Glu) is assigned to the high number of amide bonds and branched structure as a result of intense crosslinking. The presence of pores is strongly correlated with the swelling abilities of potential hemostatic agents. All samples also have very low density, typical for aerogels (Figure 4).

### 2.3. Water Vapor Transmission Rate of the Chitosan Aerogels

Proposed hemostatic agents are dedicated especially for surface wounds. Therefore, they should not only be able to prevent high blood loss, but also promote damaged tissue regeneration. For successful skin recovery it is essential to provide appropriate conditions during the healing process, such as appropriate moisture and water vapor transmission rate (WVTR), among others. It is known, that the required WVTR value is different in the case of healthy tissues vs. wounds. Obtained results (Figure 4) show that all of the prepared aerogels have a very high WVTR which is desired in the case of damaged epidermis and dermis under recovery [19]. It can be noticed, that the parameter in most cases is correlated with the porosity.

### 2.4. Antioxidant Activity of the Chitosan Aerogels

**Figure 5:**
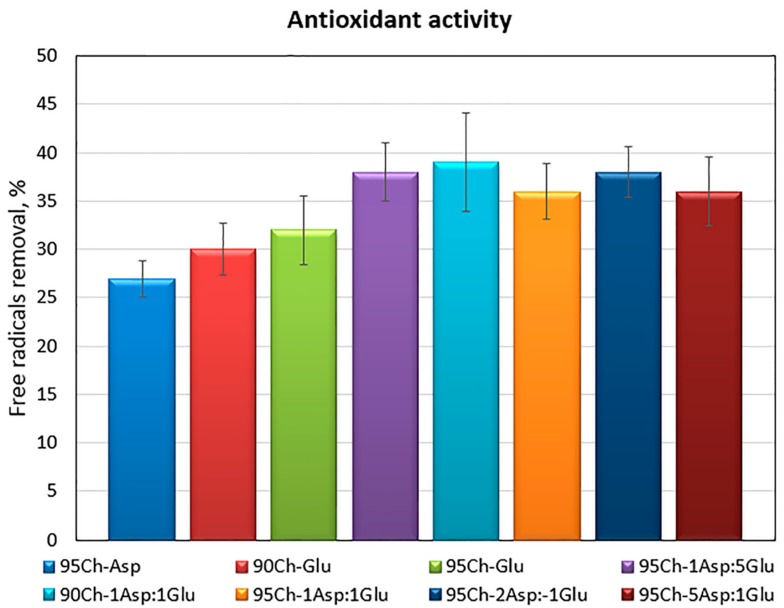
Antioxidant activity of the aerogels prepared via the crosslinking process using glutamic and aspartic acid against DPPH free radicals.

During any tissue damage oxidative stress may occur and free radicals are generated. It may lead to cell apoptosis due to proteins and genetic material damage [26,27,28]. Therefore, to protect biological macromolecules antioxidants should be applied. Chitosan is known for its antioxidant activity which can be assigned to free amino groups as well as pyranose rings [26,27,28]. However, chemical modification may have a negative impact on this property due to the decrease of free NH_2_ groups. Figure 5 presents results of the antioxidant activity of the samples against DPPH (2,2-diphenyl-1-picrylhydrazyl) radicals. One may observe, that all of the investigated aerogels had the ability of free radical scavenging and this property is correlated with the crosslinking agents—the samples crosslinked with two amino acids have significantly higher antioxidant capability. Such results can be explained by the high number of free amino groups coming from both aminoglucose mers of chitosan as well as incorporated and grafted amino acid chains [28]. The results suggest that proposed biomaterials can have a protective effect on biomolecules and cells.

### 2.5. In Vitro Degradation and Biodegradation Study

**Figure 6:**
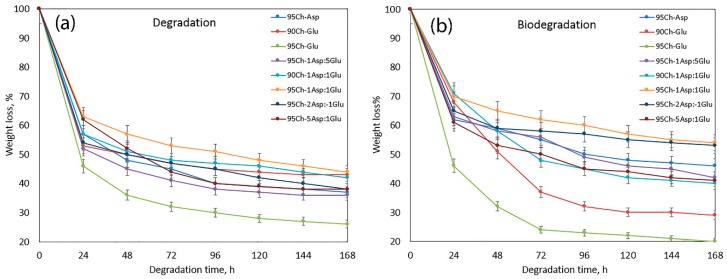
(**a**) In vitro degradation of the hemostatic agents in sterile simulated body fluid (SBF); (**b**) in vitro biodegradation study in SBF containing human lysozyme.

Proposed hemostatic agents are dedicated to various applications. They may be used during surgery to prevent blood loss or they can be applied on superficial and deep wounds to stop hemorrhaging and promote the healing process. Therefore, they should biodegrade to non-toxic substances during this short period of time. Pure chitosan is biodegradable under in vitro and in vivo conditions by enzymes which break glycosidic bonds [21,22,25]. However, its chemical modification resulting in the formation of new chemical bonds as well as incorporation of various crosslinkers, photoinitiators and other molecules may significantly hamper this property and cause cytotoxicity and pyrogenic effects due to local pH decrease, among others. Figure 6a presents results of a degradation study run in simulated body fluid (SBF) for seven days, whereas Figure 6b shows the results of biodegradation with lysozyme. One may observe that the highest (bio)degradation rate occurs during the first 24 h. It can be noticed that all of the evaluated samples are biodegradable up to 80% for one week. What is interesting is that there is no evident correlation between samples’ chemical composition and susceptibility to degradation. It can also be noticed, that aerogels decompose in a similar manner in both environments.

### 2.6. FT-IR Study

**Figure 7:**
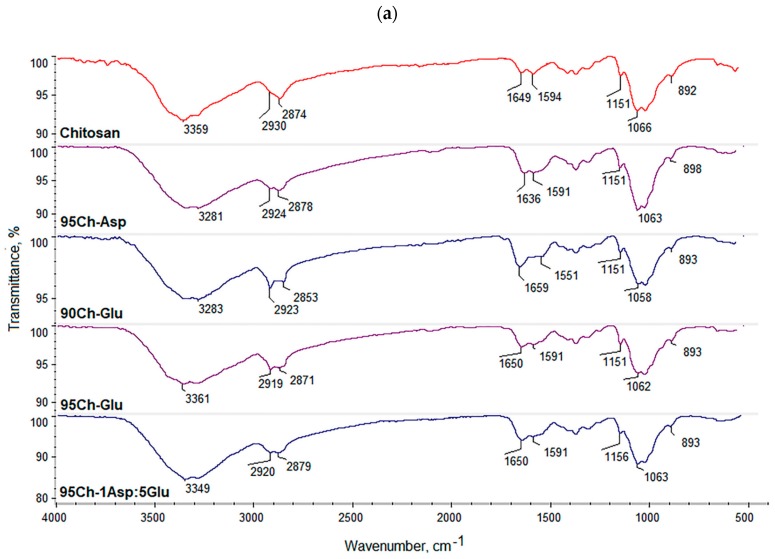

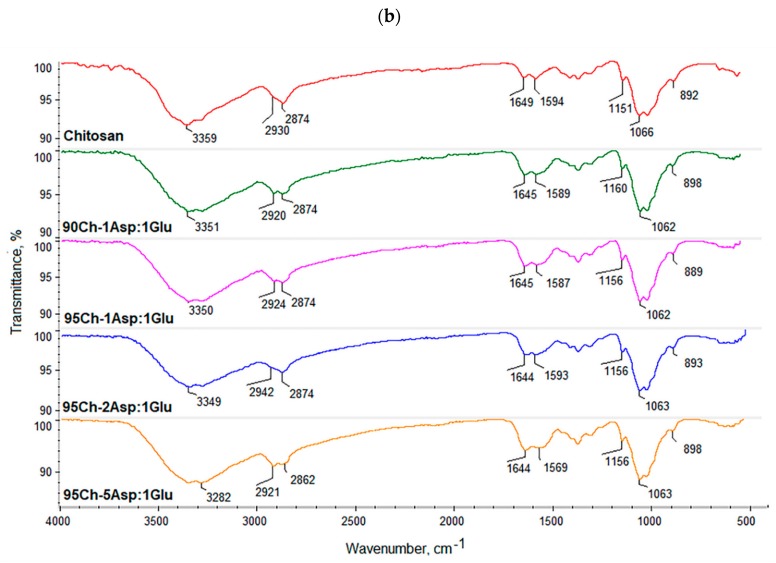
(**a**,**b**) FT-IR spectra of the samples after biodegradation.

As shown at Figure 6a,b, all of the samples lost their weight even up to 80%. The FT-IR spectra (Figure 7a,b) show that the weight loss occurred as a result of amide bonds hydrolysis in the first place as well as an enzymatic break of β-glycosidic bonds between chitosan mers. However, it seems that lysozyme activity did not have a significant impact on biomaterials disintegration during the first days of biodegradation. The FT-IR study showed that in the first stage of biodegradation, incorporated amino acids are removed from the polymeric backbone which confirms a significant decrease in the intensity of bands typical for amide bonds and free amino groups. At the same time the changes in the intensity of bands coming from pyranose rings as well as -O- bridges between polymeric units are very small. It can be noticed, that FT-IR spectra of hemostatic agents after seven days of incubation in SBF containing lysozyme are almost identical with FT-IR of the native chitosan. Therefore, it can be stated that biodegradation occurs in two stages. In the first one, chitosan changes its structure from branched to linear, whereas in the second stage polymer transforms into oligomers which may be incorporated into certain cell cycles as well as naturally removed from the body with other fluids. It may also be assumed that the slow release of amino acids (l-aspartic and l-alutamic acids) will not cause any significant pyrogenic states since they naturally occur in the human body and have lower acidity than, for example, lactic acid. The obtained data demonstrate that all samples are biodegradable and may be applied in vivo during surgeries without the necessity of reoperation of the patient after some period of time.

### 2.7. Blood-Clotting Experiment and Scanning Electron Microscopy (SEM) Analysis

**Figure 8:**
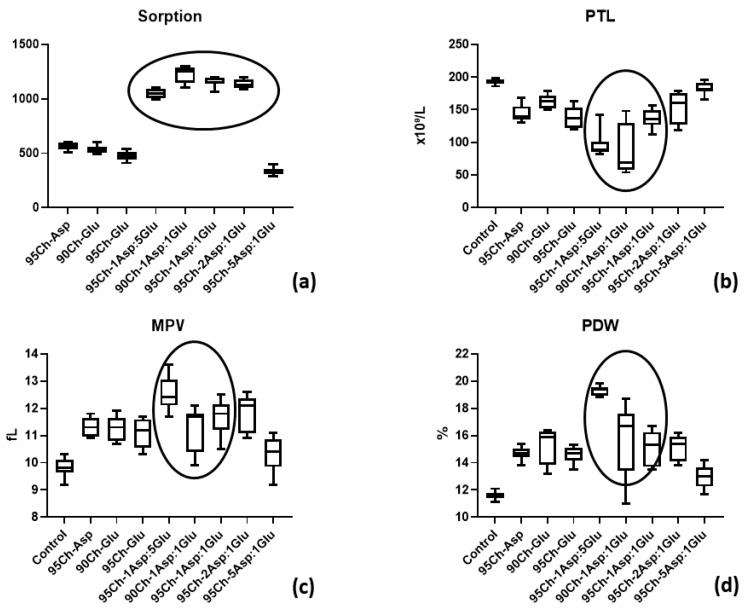
(**a)** Blood sorption by chitosan sponges and (**b**) platelet (PLT), (**c**) mean platelet volume (MPV), and (**d**) platelet distribution width (PDW) parameters after blood clotting test.

Figure 8 shows high sorption ability of all types of chitosan sponges ranging from 700% to 2400% from the initial weight. But 95Ch-1Asp:5Glu, 90Ch-1Asp:1Glu, 95Ch-1Asp:1Glu and 95Ch-2Asp:1Glu provide significantly better sorption. Ordinary one-way ANOVA test shows significant differences between sorption ability (*p* ≤ 0.0001). Blood sorption is a critical stage during the hemostasis using dressing materials and future events as well as material effectiveness strongly depend on this initial stage.

Platelet (PLT) levels after blood clotting test can show their involvement in clot formation during hemostasis. The ANOVA test demonstrates the significant difference in PLT level within all groups (*p* ≤ 0.0001). Multiple comparisons analysis shown that PLT concentration in blood after immersion of 95Ch-1Asp:5Glu, 90Ch-1Asp:1Glu and 95Ch-1Asp:1Glu sponges was significantly lower to control and other groups (*p* ≤ 0.0001 and *p* ≤ 0.001). It must be noted that PLT levels in 95Ch-5Asp:1Glu group do not significantly differ from the control (*p* = 0.79). MPV (mean platelet volume) and PDW (platelet distribution width) are parameters that depend on PLT shape that will change during the blood clotting process. Interaction of chitosan sponges with blood leads to significant increases of both parameters (ANOVA, *p* ≤ 0.0001), but MPV and PDW in 95Ch-5Asp:1Glu group did not differ from the control blood according to the multiple comparison analysis (*p* = 0.072 and *p* = 0.32). PLT parameters correlate with their number in blood after clotting—the same 95Ch-1Asp:5Glu, 90Ch-1Asp:1Glu, 95Ch-1Asp:1Glu sponges lead to significant MPV and PDW elevation. The results show that proposed biomaterials have higher hemostatic activity when compared to pure chitosan [2].

**Figure 9:**
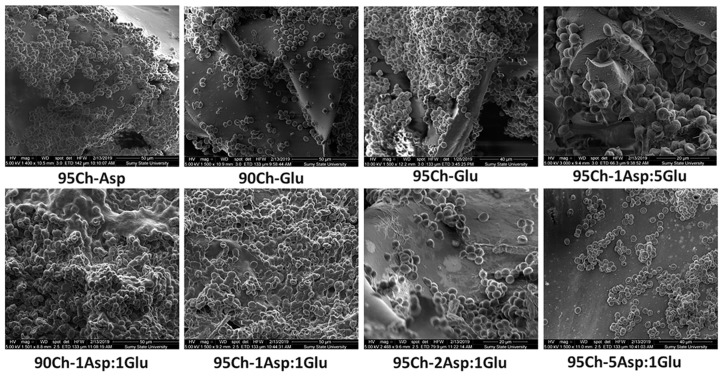
Scanning electron microscopy of chitosan sponge after blood clotting test with adhered blood cells.

SEM shows (Figure 9) blood cell distribution over the sponges, but the number of cells is different and dependent on sponge type. Blood cells mostly completely cover 95Ch-Glu, 95Ch-1Asp:5Glu, 90Ch-1Asp:1Glu and 95Ch-1Asp:1Glu samples and penetrate to the pores. Other samples gave less cells that form separate aggregates and did not firmly connect with the sponge’s surface. We can see soft substance over the cells in 95Ch-1Asp:5Glu, 90Ch-1Asp:1Glu and 95Ch-1Asp:1Glu samples that are probably blood proteins, including thrombin that is the main component of a blood clot. We can observe that this substance makes a connection between cells and sponge. The results of the cells adhesion are superior to those obtained with pristine chitosan [2]. The results correspond to the data collected by other researchers [31,32,33].

### 2.8. Cell Toxicity Experiment

**Figure 10:**
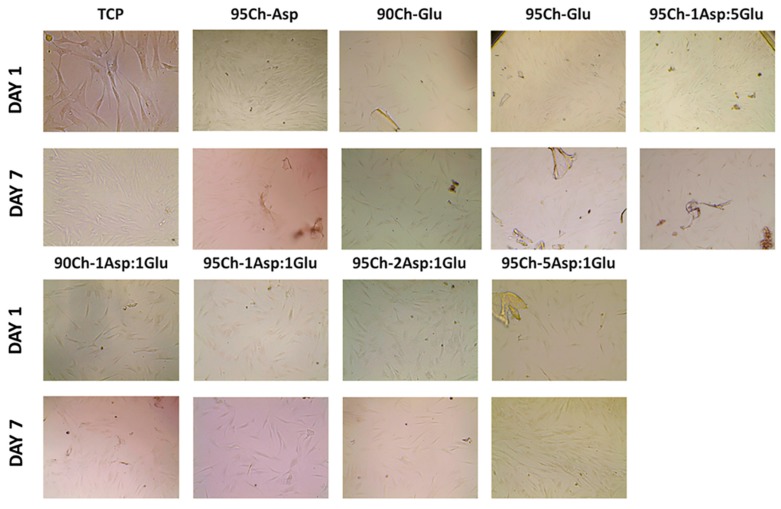
Optical image of human cell primary fibroblasts at one and seven days after cultivation with chitosan sponges. (TCP—tissue culture plastic). Magnification X200.

The main requirement for all materials for biomedical application is the safety and absence of adverse effects. Cell culture experiment in human primary fibroblasts showed an absence of cell toxicity during the seven days of cultivation. Material degradation during the experiment and polysaccharide release leads to media coloring of the fluorescent assay; as a result, we were unable to obtain a resazurin measurement. During the experiment we analyzed cell morphology and confluence. We can see confluent cell culture one and seven days after sponge immersion (Figure 10). Initial cell confluence was from 40% to 50% of the culture well, increasing up to 65–75% in all experimental groups and control (tissue culture plastic (TCP)). Some cells directly attached to the remnant of sponges with no morphological changes. Cell viability does not depend on chitosan sponge type. The lack of cytotoxicity corresponds to previously obtained results on L929 mouse fibroblasts [10,35].

### 2.9. Antibacterial Properties

All tested sponges demonstrate antibacterial activity against both types of microorganisms. The summarized results are shown in Table 1 and Table 2.

The time-kill kinetics profile of sponges against *E*. coli showed a reduction in the number of viable cells over the first 2, 4 and 6 h of incubation, followed by a whole killing of tested microorganisms. The most effective samples against *E. coli* were sponges with the following formulations: 95Ch-Glu, 95Ch-2Asp:1Glu and 95Ch-5Asp:1Glu which caused total decontamination of the media in 2 h.

In contrast, the antimicrobial activity of the chitosan sponges against *S. aureus* was not so unambiguous. Sponges 95Ch-Glu and 95Ch-1Asp:1Glu demonstrated the best bactericidal activity and all germs were killed within 2 h of incubation. 95Ch-5Asp:1Glu sponge initially demonstrated weak antibacterial effectiveness with minor increasing in the bacteria number during 6 h of incubation and total killing of microorganism in 8 h. At the same time all other formulations showed lesser inhibitory actions against *S. aureus* comparatively to *E. coli*. Mild suppression of bacterial multiplication in the first 4 h of incubation was changed to a sharp increase in microbial concentration in the cultivation media with maximal concentration of microorganism after 24 h of incubation. Antibacterial activity is a highly desired property in the case of hemostatic agents dedicated to use under non-sterile conditions. Presented data show that proposed biomaterials are superior to those containing iodine ions [8] or antibiotics [19] and others [24] since they can fight pathogens without the necessity of applying additional biocidal substances which may have a negative impact on patients’ health.

## 3. Materials and Methods

For the hemostatic agents, chitosan with 90% and 95% deacetylation degree was used and prepared from shellfish. l-aspartic acid, l-glutamic acid, 1,2-propanodiol, ethanol 95% and human lysozyme were purchased from Sigma Aldrich, St. Louis, MO, USA. For the sterile simulated body fluid preparation NaOH, NaCl, NaHCO_3_, KCl, KH_2_PO_4_·3H_2_O, MgCl_2_·6H_2_O, CaCl_2_ and Na_2_SO_4_ were used, and which were also purchased from POCH, Gliwice, Poland. To provide SBF sterility the solution was filtered using 0.2 µm filter. *Escherichia coli* B 926 and *Staphylococcus aureus* B 918 obtained from the National Collection of Microorganisms (D. K. Zabolotny Institute of Microbiology and Virology) were used in the experiment. All bacteriological media were purchase from HiMedia (Maharashtra, India) and resazurin assay was from from Sigma-Aldrich (Taufkirchen, Germany). For the cell culture study all media and reagents were purchased from Gibco^®^, Gaithersburg, MD, USA. Human dermal fibroblasts (HDFs) were obtained from the medical company Ilaya (Kyiv, Ukraine).

### 3.1. Chitosan Hemostatics Synthesis

All chitosan aerogels were obtained under microwave-assisted conditions using a household microwave according to Green Chemistry principles. For the synthesis, each time 0.5 g of chitosan with 90% or 95% deacetylation degree (DD) was dissolved in the aquatic solution of amino acid: l-aspartic, l-glutamic or a mixture thereof (Table 3). After 30 min, 10 mL of propylene glycol was added. Ready homogenous solution was placed in a reaction vessel and subjected to microwave radiation for 1 min until complete water evaporation (power = 900 W). Then, crosslinking reaction was performed for 2 min (power = 900 W), where propylene glycol served as a high boiling solvent. Obtained hydrogels were swollen with distilled water and washed out until pH = 7 from unreacted acids. After that hydrogels were lyophilized and transformed into aerogels.

### 3.2. Fourier Transform Infrared Spectroscopy (FT-IR) Analysis

All FT-IR/ATR (Attenuated Total Reflectance) analyses were performed using IR Thermo Nicolet Nexus X 470 spectrometer (diamond crystal ATR), Waltham, MA, USA. The range was between 400 and 4000 cm^−1^ with 32 scans and 4 cm^−1^ resolution.

### 3.3. Porosity and Density Study

The density and porosity of the obtained chitosan materials were determined by isopropanol displacement because it does not wet the sample. Investigated biomaterials were placed into the previously measured volume of isopropanol. After a fixed time (5 min) the change in volume of the alcohol-impregnated aerogel was measured. Then the studied chitosan scaffold was removed from the isopropanol. In the last step the difference in isopropanol volume was measured. Based on the obtained data, density (Equation (1)) and porosity (Equation (2)) were calculated using the following equations:

d = W/(V_2_ − V_3_) (1)

p = (V_1_ − V_3_)/(V_2_ − V_3_) ∙ 100% (2)

where:

d—density, g/cm^3^
p—porosity, %
W—weight of the investigated sample, g
V_1_—initial volume of isopropanol, cm^3^
V_2_—volume of isopropanol with immersed sample, cm^3^
V_3_—volume of isopropanol after sample removal, cm^3^

All experiments were repeated 3 times (*n* = 3). 

### 3.4. Water Vapor Transmission Rate

To determine the water vapor transmission rate (WVTR) samples were fixed onto the opening (area 1 cm^2^) of the polystyrene well using polymeric glue. Each plate contained 5 mL of distilled water. The studies were carried out for 24 h at 37 °C. The WVTR was measured basing on the amount of water loss. Water vapor transmission rate was calculated using the following equation:

WVTR = (W_t_ − W_0_)/(tA)(g·m^−2^·d^−1^) (3)

where:

W_0_—the initial weight,
W_t_—the weight after time t,
t—the measuring time
A—the area of the opening of the polystyrene well

The experiments were repeated 3 times (*n* = 3).

### 3.5. Antioxidant Activity

Antioxidant properties of the prepared chitosan scaffolds were investigated by a standard DPPH method. For this purpose, a solution of DPPH in methanol was prepared so that the solution absorbance was 1.0 at 517 nm using an Aligent 8453 spectrophotometer. To determine the ability of free radicals scavenging, 0.10 g of each sample was placed in 5 mL of DPPH solution (25 mg/L) and left in darkness for 1 h with constant shaking. Then, the absorbance of each solution was measured at 517 nm. The percentage of the free radicals removed was calculated using Equation (4):

%S = (A_s_ − A_c_)/A_c_ (4)

where:

%S—the % of the free radicals which were neutralized
A_c_—the absorbance of the DPPH solution without the sample
A_s_—the absorbance of the DPPH solution containing the sample

All experiments were repeated 3 times (*n* = 3). 

### 3.6. In Vitro Degradation and Biodegradation Study

Chitosan hemostatic degradation study was performed in simulated body fluid (SBF). For the experiments, the biomaterials were sterilized using an autoclave. The studies were performed for seven days. For this purpose, weighed chitosan samples where immersed in 50 mL of sterile SBF solution. The aerogels were taken out, washed with distilled water followed by drying and weighing at fixed time intervals. 

In vitro biodegradation study was conducted using human lysozyme—an enzyme which naturally occurs in the human body in tears and serum at the concentration of 7–13 mg/L. Lysozyme is an enzyme hydrolyzing β-glycosidic bonds. For the study, weighed chitosan aerogels where immersed in pure SBF and SBF containing lysozyme (concentration = 10 mg/L at 37 °C) to imitate natural conditions. The samples were taken out, washed with distilled water, dried and weighted at fixed time intervals. The percentage of degradation and biodegradation was calculated using the following equation:

(B)D = (W_0_ − W_t_)/W_0_ ∙ 100% (5)

where:

(B)D—(bio)degradation degree, %
W_0_—initial weight of the analyzed sample, g
W_t_—sample weight after time = t, min

All experiments were repeated 3 times (*n* = 3).

### 3.7. Blood Clotting Tests

Four human subjects volunteered to have 100 mL of blood drawn by a registered nurse at the Medical Institute of Sumy State University. The study was previously approved by the Ethic Committee on Medical Research of Medical Institute Sumy State University. An appropriate informed consent was obtained from all volunteers.

The strips of chitosan material weighted 40 mg were placed in individual Becton Dickinson Vacutainers^®^ each filled with 2 mL of human blood. During the next 10 min vacutainers were shaken constantly in order to provide the interaction between sponge and blood. All samples were removed, weighted and blood sorption (BS) rate was calculated as follows:

BS = W_2_ − W_1_ (6)

where:

W_1_—initial weight (40 mg)
W_2_—weight after the blood clotting test, mg

The remaining blood was used for a completed blood count (CBC) test for the study of thrombocyte adhesion and aggregation. The CBC test was performed in Medical Centre “Floris”. CBC was carried out on the hematology analyzer CELL-DYN 3700 (ABBOTT, Irving, TX, USA) using reagents DIAGON (Budapest, Hungary). The following parameters were evaluated: platelet count (PLT, ×10⁹/L), platelet distribution width (PDW, %) and mean platelet volume (MPV, fL).

### 3.8. Scanning Electron Microscopy (SEM)

Sponges were removed from blood, placed in 2% glutaraldehyde for 2 h, then dehydrated samples were placed in ethanol and dried. To avoid surface charge accumulation in the electron-probe, samples were covered with a thin (30–50 nm) layer of silver in the vacuum set-up VUP-5M (SELMI, Sumy, Ukraine). The SEM images of sponges were observed by FEI Inspect S50B (FEI, Brno, Czech Republic) with the Everhart–Thornley secondary electron detector.

### 3.9. Cell Culture

The cells were grown in 75 cm^2^ tissue culture flasks under standard culture conditions with 5% humidified CO_2_ in the air at 37 °C with medium renewal every 2–3 days. Dulbecco’s Modified Eagle Medium/Nutrient Mixture F-12 (DMEM/F-12) with L-glutamine contained 100 units/mL penicillin, 100 µg/mL streptomycin, 2.5 µg/mL amphotericin B, 10% fetal bovine serum and 1.0 ng/mL bFGF (basic Fibroblast Growth Factor). HDF were seeded in 24-well plates (1.0 mL/well) at a cell density of 5 × 10^4^ cells per well and pre-incubated overnight. Thereafter in each well the chitosan strip weighed 50 mg was added. The well with no chitosan sponge was used as a positive control (TCP—traditional cell culture on plate). Cell growth and proliferation were assessed using EVOS XL Core cell imaging system (ThermoFisher Scientific, Waltham, MA, USA). Resazurin or tetrazolium assays were unable to be carried out due to medium optical density change after interaction with chitosan [36].

### 3.10. Bacteriology Assay

Antibacterial activities of chitosan sponges were assessed against Gram-positive (*S. aureus*) and Gram-negative (*E. coli*) bacteria. Before the test the sponges were cut into small pieces (4 mg) and sterilized under UV light. The bacterial cultures were incubated in nutrient broth overnight. Next the cultures were diluted with cultivation media to the microorganism concentration equivalent to 10^4^ colony forming unit (CFU)/mL (4 log10 CFU) that was used as an inoculum.

Under an asepsis operation condition, each sample was placed in the tubes with 2 mL of microbial inoculums and incubated for 2, 4, 6, 8, 10 and 24 h at 37 °C. The tubes containing growth medium and tested samples without inoculums were used as controls. After that 100-μL aliquots from tubes were spotted onto plates with solid media and incubated at 37 °C for 24 h. Viable organisms were counted in log10. All tests were conducted in triplicate.

## 4. Conclusions

The aim of the following research was to develop a new type of chitosan-based hemostatic agents. As a result of the performed syntheses, crosslinked aerogels were obtained due to the formation of amide bonds between chitosan and amino acids without the application of acetic acid. The use of l-aspartic and l-glutamic acids prevented the loss of free amino groups responsible for favorable chitosan biological properties. The biomaterials had high porosity and very good antioxidant properties. They also enabled water vapor permeability. The products were susceptible to biodegradation under human body-like conditions. Chitosan-based aerogels had excellent hemostatic properties which were confirmed by blood clotting tests. The SEM analysis showed that biomaterials are bioactive and interact with the blood cells which adhere to their surface thus promoting clot formation. Also, the samples were non-cytotoxic to human primary dermal fibroblasts. Moreover, investigated samples exhibited antibacterial activity against both Gram-positive and Gram-negative bacteria strains. Altogether, the proposed biomaterials have great potential in the area of blood loss prevention and may constitute a promising alternative to commercially available biomaterials. The modification strategy enabled preparation of the materials with unique biological properties.

## Figures and Tables

**Table 1:** Time-kill kinetics of chitosan sponges against *E. coli.*

Time of Incubation (hours)	Sample/Isolated Microorganisms in Log (CFU—Colony Forming Unit)							
	95Ch-Asp	90Ch-Glu	95Ch-Glu	95Ch-1Asp:5Glu	90Ch-1Asp:1Glu	95Ch-1Asp:1Glu	95Ch-2Asp:1Glu	95Ch-5Asp:1Glu
**0**	4	4	4	4	4	4	4	4
**2**	2	3.7	0	2	3.5	2	0	0
**4**	2	2	0	0	2	2	0	0
**6**	0	0	0	0	2	2	0	0
**8**	0	0	0	0	0	0	0	0
**10**	0	0	0	0	0	0	0	0
**24**	0	0	0	0	0	0	0	0

**Table 2:** Time-kill kinetics of chitosan sponges against *S. aureus.*

Time of Incubation (hours)	Sample/Isolated Microorganisms in Log (CFU)							
	95Ch-Asp	90Ch-Glu	95Ch-Glu	95Ch-1Asp:5Glu	90Ch-1Asp:1Glu	95Ch-1Asp:1Glu	95Ch-2Asp:1Glu	95Ch-5Asp:1Glu
**0**	4	4	4	4	4	4	4	4
**2**	2	2	0	2	2	0	2	2
**4**	5	3	0	3	4	0	5	3
**6**	5	5	0	4.7	5	0	6.7	5.7
**8**	5	6.7	0	6.7	6.7	0	6.7	0
**10**	5	6.7	0	6.7	6.7	0	6.7	0
**24**	5	6.7	0	6.7	6.7	0	6.7	0

**Table 3:** Chitosan aerogels composition.

Sample	Crosslinking Agents		Chitosan Deacetylation Degree (DD)
	l-Aspartic (Asp)	l-Glutamic (Glu)	
95Ch-Asp	0.84 g	-	95%
90Ch-Glu	-	0.84 g	90%
95Ch-Glu	-	0.84 g	95%
95Ch-1Asp:5Glu	0.74 g	0.22 g	95%
90Ch-1Asp:1Glu	0.50 g	0.50 g	90%
95Ch-1Asp:1Glu	0.50 g	0.50 g	95%
95Ch-2Asp:1Glu	0.70 g	0.30 g	95%
95Ch-5Asp:1Glu	0.17 g	0.84 g	95%
